# In search of excellence

**DOI:** 10.1093/jhps/hnad045

**Published:** 2023-12-21

**Authors:** Richard E Field

**Affiliations:** Editor-in-Chief, Journal of Hip Preservation Surgery

## Abstract

A little while ago, I had the opportunity to spend some time in the operating room (theatre) of one of the world’s most experienced and technically skilful hip arthroscopists. How I wish I had made the effort to visit this surgeon earlier in my career and how I regret that there are still so many surgeons I have failed to visit. Every surgeon who has allowed me into their operating room has shown me something that has enhanced my practice and learning to execute their manoeuvres always adds to the pleasure of surgical practice.

Like all surgeons, hip arthroscopists discuss such topics as patient selection, how to manage patient expectations and the outcomes they seek to achieve. The challenge of patient selection for femoroacetabular impingement (FAI) surgery depends on diagnostic pathways that are addressed in Delphi consensus paper by Radha et al. [1] in this issue (JHPS 10.3–4). The authors secured consensus from 174 hip preservation surgeons on 55 statements pertaining to the assessment of patients with femoroacetabular impingement syndrome (FAIS). While the work will provide a valuable reference for surgeons seeking to confirm or exclude FAIS, the study does not provide any guidance on which symptoms and signs are the best predictors of outcome. While predictors of outcome for FAI surgery have been reported [2], testing the 55 statements against subsequent PROMS outcomes will provide valuable additional information and JHPS would welcome manuscripts addressing this question.

During my visit to see my friend undertake arthroscopic surgery, he observed that the UK Fashion study on FAIS [3] reported a mean 12-month post-operative iHOT [4, 5] score of 58.8 and the most recent Non-Arthroplasty Hip Registry (NAHR) report [6] details a mean iHOT score of 58.44. My host acknowledged that these patient groups enjoyed approximately 20- and 25-point iHOT score improvements respectively but, queried the value of a surgical intervention that provides patients with a mean iHOT score below 80 points. I have since scored myself using the iHOT-12 questionnaire. If an individual of my advanced years can record a score of almost 100 points, a younger individual with a score below 60 would find recreational sports challenging and my host’s concern is understandable.

Reports on case-series and cohort groups have shown that arthroscopic treatment of FAI-related intra-articular damage reduces symptoms and can return patients to activity with high patient satisfaction [7–9]. However, for the general population, a mean 80-point outcome may be an unachievable target and we may be undertaking surgery on individuals for whom there is no intervention that can achieve this goal. It is possible that once a hip has been damaged by FAI there is only so much that we can do to relieve discomfort and restore function. If this is the case, patients may be grateful for the limited benefit that surgery can offer. Alternatively, we may be failing to identify the subset of patients for whom surgery can achieve the mean 80-point target and failing to select the most appropriate patients for surgery.

Another possibility is that some surgeons can consistently deliver significantly better outcomes through more effective surgery. If this is the case, we should identify what these surgeons do, how they do it and find ways to help other surgeons emulate their results. Knowledge can be shared through journals and conferences, techniques can be shared by video presentations but, surgical skills are best observed in the operating room. Regular attendance in the operating rooms of our colleagues should be encouraged throughout our careers, both to proctor and observe. Having experienced the extremes of access to operating rooms around the world, I am mindful that the surgical community must overcome a myriad of administrative hurdles to ensure free exchange of our skills. Nevertheless, it cannot be beyond the compass of our national societies to create ‘Surgeon Accreditation Passports’ that would better enable us to visit hospitals both to share our expertise and observe the skills of our colleagues.

A recent Newsweek report on the World’s Best Specialized Hospitals 2024 [10], ranked the New York, Hospital For Special Surgery as No 1. It is from this institution that we have included a paper looking at the effect of variation in intraoperative traction time on a range of PROMS outcomes scores [11]. The principle finding of the paper was that longer traction times do not adversely affect patient outcomes. It is also noteworthy that in this study, the mean one and 2-year post-operative iHOT scores were 77.2 and 79.3, respectively. These results support my friend’s contention that a mean post-operative iHOT score of 80 is achievable.

Finally, the combined release of Issues 9.3 and 9.4 provides the opportunity for a guest editorial from members of the UK NAHR team [12]. Christian Smith, Vikas Khanduja and Ajay Malviya reflect on the registry’s evolution through its first decade and the increasingly valuable information provided in each annual report. Securing the engagement of all the UK’s hip preservation surgeons remains a challenge; as does maintaining patient engagement after surgery. Unlike the UK National Joint Registry (UK NJR) [13] submission cases to the UK NAHR remains voluntary and the authors make a strong case for this to be made compulsory. It would be equally helpful if patients could be persuaded to provide feedback that matches the determination of their surgeons.
