# Correction to: In search of excellence

**DOI:** 10.1093/jhps/hnae024

**Published:** 2024-06-26

This is a correction to: Richard E Field, In search of excellence, *Journal of Hip Preservation Surgery*, Volume 10, Issue 3–4, August/December 2023, Pages 133–134, https://doi.org/10.1093/jhps/hnad045

The following changes have been made to the originally published manuscript.

The section **Data Availability** and contents have been removed from the article.

